# Impact of dendritic cells and adjuvant on the in vivo immunogenicity of HTLV-1 Tax 11-19 epitope in hybrid HLA-A2.1/DTR transgenic mice

**DOI:** 10.1186/1742-4690-11-S1-P72

**Published:** 2014-01-07

**Authors:** Divya Sagar, Shet Masih, Todd Schell, Saifur Rahman, Pooja Jain, Zafar K Khan

**Affiliations:** 1 Drexel Institute for Biotechnology and Virology Research, and the Department of Microbiology and Immunology, Drexel University College of Medicine, Doylestown, PA, USA; 2 Department of Microbiology and Immunology, Pennsylvania State University College of Medicine, Hershey, Pennsylvania, USA

## 

Individually, HLA-A2.1 and CD11c-DTR transgenic mouse strains have been used to investigate the immunopathogenesis of different viruses and have provided important insights into current understanding of host-pathogen interactions during human T-cell leukemia virus type 1 (HTLV-1) infection. Here, these mice enabled the study of the CD8 T-cell immune response against a known MHC class I HLA-A2.1-restricted epitope 11-19 of the viral oncoprotein Tax delivered along with tetanus helper peptide without or with incomplete Freund’s adjuvant (IFA) in the absence and presence of dendritic cells (DCs). First, a cross breeding strategy was utilized to generate a HLA-A2.1/DTR hybrid strain that carries an HLA-A2.1 gene and diphtheria toxin receptor gene for in vivo depletion of CD11c^+^ DCs. Upon in vitro stimulation of splenocytes from immunized mice with autologous bone marrow-derived DCs primed with Tax11-19 antigen, DC-depleted mice showed marked attenuation in the proliferation of CD8^+^ T-cells when compared with the non DC-depleted mice. Additionally, mice immunized with adjuvant demonstrated a much higher frequency of Tax11-19-specific cells response but overall reduced proliferation compared to the non-adjuvant group. A significantly high serum level of IL-6 coincided with depletion of DCs while a decrease in TGF-β cytokine associated with adjuvant use irrespective of DCs’ presence. In conclusion, these studies not only demonstrate that the clinically characterized Tax epitope 11-19 can be a potential candidate for the DC-based anti-HTLV-1 vaccine but also illustrate a broader application of the new HLA-A2.1/DTR-transgenic hybrid mouse strain as an important tool to investigate DC involvement in human class-I-restricted immune responses.

